# A Triplet Parallelizing Spiral Microfluidic Chip for Continuous Separation of Tumor Cells

**DOI:** 10.1038/s41598-018-22348-z

**Published:** 2018-03-06

**Authors:** Hongmei Chen

**Affiliations:** 1 School of Mathematics and Physics of Science and Engineering, Anhui University of Technology, Maanshan, 243002 China; 2 Division of Nanobionic Research, Suzhou Institute of Nano-Tech and Nano-Bionics, Chinese Academy of Sciences, Suzhou, Jiangsu 215123 China

## Abstract

Inertial and deformability- based particles separations gradually attract more significant attentions. In this work, we present a hybrid chip by combining the advantages of inertial and deformability –based principle. The chip is a triplet parallelizing spiral inertial microfluidic chip interconnected with numerable tilted slits (Spiral-Slits Chip) for continuous separation of circulating tumor cells. Utilizing the inertial lift and viscous drag forces, different sized particles achieve different equilibrium at distinct streamlines of the spiral microchannel. Numerable tilted slits are organized along the flow direction. They frequently transport segregated streamline particles into a paralleled smaller microchannel. These frequent dragging results in the amount of certain sized particles in the original microchannel gradually and dramatically reduced. Inertial separation of distinct sized particles could be achievable. Two arrays of numerable tilted slits function as bridges. This Spiral-Slits Chip could substitute for Red Blood Cells Lysis (RBCL) and is most effective for ultra-high throughput. The overall arrangement of this triplet parallelizing spiral inertial microfluidic reflects stable streamlines distribution in the first main microchannel. Combining with Ellipse filters, robust and reproducible capture of CTCs could be achieved at high flow rates. Optical absorption detection has been tentatively tested, and this could simplify the process.

## Introduction

Separation of microparticles has critical significance for clinical enumeration in micro/nano-manufacturing^1^, biological molecular analysis^2^, and medical diagnosis^3–6^. Size and deformability -based filtration could efficiently segregate physically different sized particles. Currently, wide application has been applied to isolate Circulating tumor cells (CTCs, 15 µm–25 µm) from peripheral blood (9 µm–16 µm for white blood cells, WBCs and 4 µm-8 µm for red blood cells,RBCs).

Theoretical and numerical analysis of deformability-based circulating tumor cells (CTCs) isolation has been developed ^7–11^. Towards the cross-section optimization, five different cross- sections (circular, square, triangle, and two rectangles with different aspect ratio) are numerical studied and compared with theoretical quasi-static design method^7^. Towards the operation optimization, two critical velocities were analytical derived, a critical velocity with highest critical pressure^8^ and a critical velocity with minimum impulse transportation^10^. Model for predicting the extra pressure of separating a simple CTC^11^ and a compound CTC^12^ are expressed. These results may guide future CTC filter designs.

Experimentally, by designing well-organized particular microstructures such as micropores, micropillars or microfilters on the microfluidic chip, isolation could be achievable. In 2000, Giovanna Vona performed an assay of isolation by size of epithelial tumor cells (ISET) with 8-µm-diameter cylindrical pores^13^. Masahito Hosokawa presented a size-selective microcavity array to produce a negative pressure to entrap tumor cells^14^. Swee Jin Tan proposed a crescent well consisting of three microposts with 5 µm gap to trap tumor cells^15^. Sarah M. McFaul proposed a microfluidic funnel ratchet with series of arrays reduced from 15 µm to 4 µm to discriminate CTCs^16^. Most of these techniques could not avoid clogging problem. Red blood cells lysis (RBCL) is usually required for pre-processing^17^. Prolonged sample processing would be induced by high fluidic resistance from microposts barriers, endured prepossessing and staining process before immunofluorescence. Pre-processing, clogging, and limited throughput are bottlenecks of those approaches and are emergently improved.

Based on our survey, almost all clinical experiments are related to immunofluorescence, fixation and permeabilization, staining, flushing, and detection. It would take more than one hour to complete this long process. Including RBCL, several hours would be spent to perform supplementary processing in one clinical experiment. It is really emergent necessary to simply those assistant procedures with advanced micro/nano technology and intuitively optical absorption spectroscopy. Since every material has its own characteristic spectroscopy, this is theoretical principle background to recognized CTCs or tumor cells from hematological cells. Replacing with optical option and processing at high throughput such as utilizing inertial chip are in need.

Inertial focusing could separate and concentrate particles at high throughput^14^. Structures are in the shape of straight^18^, serpentine^3,19,20^ and spiral^21,22^. There are many applications of inertial focusing such as separation of circulating tumor cells^1^^23^ and isolation of blood components^24^. A strategy of spiral microfluidic devices utilizes hydrodynamic forces for high throughput size-based continuous separation. In CTCs isolation, a spiral biochip with a rectangular cross-section could process metastatic non-small cell lung cancer (NSCLC) at a flow rate of 3 mL/h^25^. With a trapezoid cross-section, the flow rate could be enhanced to 7.5 mL of red blood lysed blood in about 8 min from the peripheral blood of patients with metastatic breast and lung cancer^26^. A cascaded spiral microfluidic device could continuously separate CTCs with high purity^27^. Two subchannels are arranged at bifurcating regions. The flow rate for those assays is controlled at 550 µl/min. Average separation efficiency could reach 86.76% with a 97.91% leukocyte depletion rate, and >90% cells remained viable.

A notable thing that needs to pay attention is the resolution. Although inertial chip could be designed to process CTCs, processing is limited in resolution. Separated CTCs is mixed with blood constituents. CTCs could not be separated directly, and throughput is needed to be improved. The mixture of low purity would block further molecular genetic analysis such as Reverse Transcription-Polymerase Chain Reaction (RT-PCR). However, if we could separate all CTCs, almost whole CTCs in one sample mixed with certain hematological cells, this could be utilized as preprocessing as RBCL. Combining with physical-based filters, that amount of blood cells could be removed through filtering easily. Due to processing of breaking blood cells on ice and centrifuging twice, RBCL will take more than half an hour and transfer of tubes causes possible CTCs loss. Filtering through inertial at higher throughput would work perfectly as an exquisite preprocessing microfluidic chip substituting for RBCL. Focusing and concentration of whole CTCs in one microchannel would realize CTCs enrichment. This is the direction we will explore toward.

**Figure 1:**
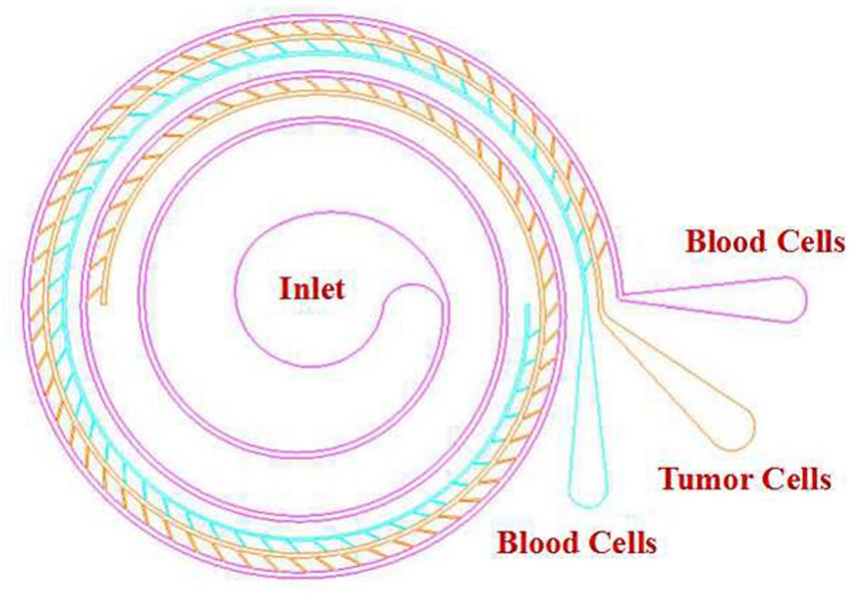
Schematic of the triplet-microchannel spiral microfluidic chip with numerable tilted slits (Spiral-Slits Chip) for separation of tumor cells. There are three main microchannels composing the spiral microfluidic chip. One is shorter than the adjacent microchannel. These three microchannels are interconnected by two arrays of numerable tilted slits transporting streamline cells from one main microchannel into the other along the flow direction. Inlet is located in the center. Actually, there is only a small hole with a diameter of 1 mm used to load cell suspension with tumor cells into the triplet-microchannel spiral microfluidic chip (Spiral-Slits). After inertial circular spin, tumor cells flow out from the middle outlets, and blood cells flow out from the outside and inside outlets with 1 mm diameter of the two holes.

**Figure 2:**
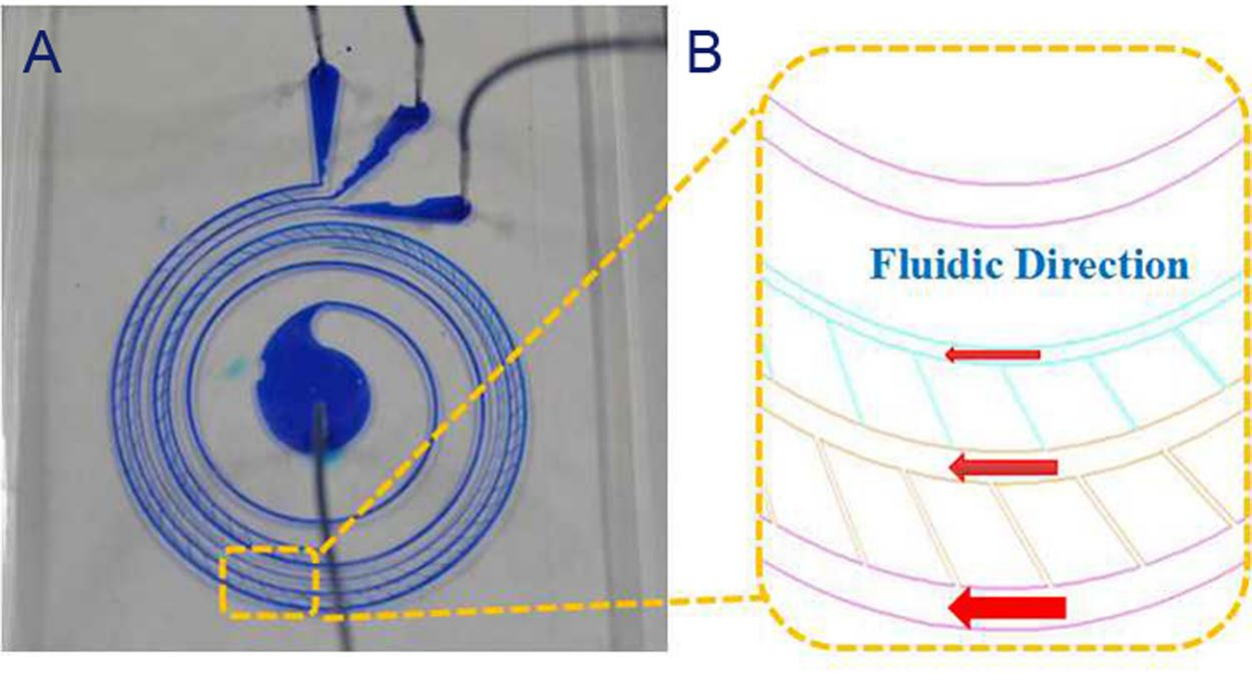
Schematic and detailed structure of the centrifugal-based differential device (Spiral-Sits Chip) (**A**) Schematic illustrating of a triplet-microchannel spiral microfluidic chip (Spiral-Slits Chip). Fluid could flow fluently inside the triplet-microchannel spiral microfluidic chip (Spiral-Slits Chip) at a high flow rate. (**B**) The detailed structure of the triplet parallelizing spiral inertial microfluidic chip (Spiral-Slits Chip) with three main microchannels and numerable tilted slits interconnecting two adjacent microchannels. Tilted slits are along the fluidic direction with a certain degree of around 60°.

In this work, we present a triplet-microchannel spiral inertial microfluidic chip interconnected with numerable tilted slits (Spiral-Slits Chip) for separation of tumor cells as shown in Fig. 1. The characteristic of this separation lies in the physical difference of size between tumor cells and blood constituents^17^. Under the influence of Dean forces and inertial forces, different sized-particles would equilibrate at different positions of forming distinct particle streamlines. Big particles flow neighboring to the center of the microchannel and small particles stream along both sides of the microchannel. Numerable tilted slits at an angle of around 60° to the flow direction are arranged connecting two adjacent microchannels (Fig. 1). They function as a bridge to transport certain sized-particles from outside to inside microchannel to realize separation. Three microchannels are gradually narrowed down. A fast second or third similar inertial separation could be performed (Fig. 2). Two sized- numerable tilted slits are arranged at a certain angle to the flow direction to convey tumor cells and blood cells to two corresponding main microchannels with smaller radii, respectively. Therefore, CTCs or tumor cells are completely concentrated in the middle microchannel. Blood cells occupied the other two most and some in the middle microchannel. Optical absorption spectra have been tested to perform cell separation instead of traditional immunofluorescence method. This simplifies staining and is intuitive for detection and characterization. The performance of this triplet parallelizing spiral inertial microfluidic chip (Spiral- Slits Chip) has been evaluated with tumor cells mixed with whole blood.

## Separation principle

### Background Theory

Under inertial, the larger particle tends to form a streamline along the channel wall and small particles stream close to the inner channel. Eventually, different sized-particles formed several streamlines moving paralleled along the microchannel. High purity and fine resolution for easy detection are further needed to be improved.

In straight cylindrical Poiseuille flow, particles migration is governed by two primary inertial lift forces perpendicular to the direction of the flow. Equilibrium positions of particles are also determined by two forces. A shear-induced lift force (Fs) is induced by the parabolic velocity gradient pointing to the microchannel wall. A sustaining wall lift force (Fw) acting on the particles cause particles migrating toward the microchannel center. Under the influence of these combined two forces, migrating particles approximately equilibrate at 0.2D away from the wall with D as the diameter of the cylindrical pipe. The situation would be complicated for square or rectangular cross-section. The net inertial lift force (F_L_) is given as following^28^:

1 $${F}_{L}=\frac{\rho {U}_{m}^{2}{a}_{p}^{4}}{{D}_{h}^{2}}{C}_{L}$$

ρ is the fluid density (kg m^-3^), U_m_ is maximum fluid velocity (m s^-1^) as *U*_*m*_ = 1.5 × *U*_*avg*_, U_avg_ is the average fluid velocity (m s^-1^), a_p_ is particle diameter, D_h_ is the hydraulic diameter, D_h_ = 2wh/(w + h) (a channel of w width and h height), and *C*_*L*_ is non-dimensional lift coefficient functioning as Reynolds number (*R*_*c*_* = *$\rho {U}_{m}{D}_{h}/\mu$) relying on the position of particles^29^.

In the curvature microchannel structure, Dean Flow forms two counter-rotating vortices within the microchannel^3^. A dimensionless Dean number (D_e_) is given by

2 $$\begin{array}{c}{D}_{e}=\frac{\rho {U}_{m}{D}_{h}}{\mu }\sqrt{\frac{{D}_{h}}{2R}}={R}_{c}\sqrt{\frac{{D}_{h}}{2R}}\end{array}$$

The channel Reynolds number is defined as ${R}_{c}=\frac{\rho {U}_{m}{D}_{h}}{\mu }$

where µ is the fluid viscosity (Pa · s), D_h_ is the microchannel hydraulic diameter, D_h_ = 2wh/(w + h) and R is the radius of curvature (m) of the microchannel. A drag force induced by a Dean flow is given by the following,

3 $${F}_{D}\approx 5.4\times {10}^{-4}\pi \mu {D}_{e}^{1.63}{a}_{p}$$

Eventually, in curved microfluidic channels, the equilibrium position relies on a Dean coupled inertial migration of particles from a combination of inertial lift force (F_L_) and Dean force (F_D_).

Under the simple inertial law, separated big particles always tend to move along outside microchannel wall. For the spiral microfluidic chip, sustaining wall lift force (Fw) occupies and increases for impelling particles especially big ones migrating toward the microchannel center for a high flow rate. Shear-induced lift force (Fs) pointing to the microchannel wall is weakened at higher throughput. Under the combination of both forces, big particles tend to migrate along the centerline of the microchannel. This streamline migration has been reflected by the microstructures. Big particles such as CTCs come out from the middle microchannel and small particles such as blood constituents out from inner and outside microchannels. This Spiral-like microfluidic device in Fig. 2 is specialized for separation of CTCs as a preprocessing chip.

## Materials and Methods

### Fabrication

Figure 1 shows a schematic representation of the microfluidic chip. Widths for three microchannels are 400 µm, 300 µm, and 200 µm, respectively. Widths for two tilted slits are 80 µm and 45 µm, respectively. There are 83 80-µm tilted slits and 53 45-µm ones arranged, respectively. Three microchannels (inner 3, middle 2, outer 1) are interconnected by these two arrays. Circles for three microchannels are 3, 1.5, and 1, respectively. Totally Channel curvature with a radius ranging from 1 cm to 1.5 cm for circles. The height of the device is 70 µm. The structure of the microfluidic chip is created through lithography technology^17^. Patterns of the microstructure are drawn to produce a high-resolution transparency optical photomasks^17^. Inverse versions of the microstructures are fabricated on a silicon wafer as following^17^. The silicon wafer is spin-coating with a 7 µm thick AR-N 4450-10 (ALLRESIST GmbH, Germany), soft baking, UV light exposure and then post exposure baking^17^. After dry etching, Inverse versions of Spiral chip were generated on a silicon wafer. After surface treatment, through casting a liquid PDMS against the master and baking in an oven for 1 h at 80 °C^17^, a PDMS structure was fabricated with inlet and outlet punched^17^. After oxygen plasma exposure and the blanked area sealed, the PDMS structure was bonded to a glass microscope substrate^17^.

### Sample preparation

MCF-7 cells (human breast adenocarcinoma) were provided by Suzhou Institute of Nano-Tech and Nano-Bionics, Chinese Academy of Sciences^17^. Cells were cultured in Dulbecco’s Modified Eagle Medium (DMEM) (HyClone, USA) medium supplemented with 10% fetal bovine serum (FBS) (GIBCO, USA) and 1% penicillin-streptomycin (Ying Reliable biotechnology, China) and incubated in a humidified atmosphere at 37 °C with 5% CO_2_ atmosphere^17^. When cell lines were grown as adherent monolayers to 95% confluence, they were detached from the culture dishes with 0.25% Trypsin solution for two minutes^17^.

### The microfluidic system setup

The microfluidic system is composed of the triplet-microchannel spiral microfluidic chip (Spiral-Slits Chip), plastic tubing, syringes and syringe pumps^17^. A syringe pump was used to load cell suspension into Spiral chip. The syringe attached to the pump was connected to the inlet of the triplet-microchannel spiral microfluidic chip through plastic tubing^30^. An inverted phase contrast fluorescence microscope (Nikon, Japan) equipped with a high-speed CCD camera (Nikon, Japan) was used to observe. This device was fabricated in Polydimethylsiloxane (PDMS) bonded to a glass substrate^17^.

### Separation of spiked tumor cells in a triplet-microchannel spiral inertial microfluidic chip and percentage calculation

Around one thousand tumor cells spiked into diluted blood (5×) test the performance of separation. Volume is measured and number of cells is counted from three outlets, respectively. A total number of tumors coming out from each tunnel is calculated when counting the number of cells in the unit volume times the total volume.

## Results and Discussion

### Recovered percentage at lower flow rates

At lower flow rates, cell particles are difficult to form streamlines paralleling the channel tunnel. They are chaotic and stochastically selected to be transported from outside microchannel into inside. Three channels are all occupied by tumor cells and hematological particles. No obvious separation of tumor cells exists.

**Figure 3:**
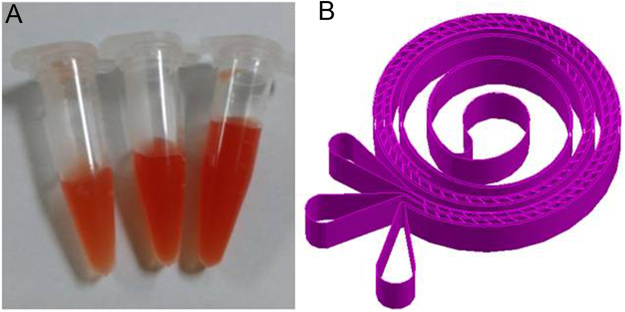
Cell suspension collected from three main microchannels and schematic diagram of the triplet-microchannel spiral microfluidic chip interconnected with numerable tilted slits (Spiral-Slits Chip). (**A**) Cell suspension collected from three main microchannels at the flow rate of 9 ml/h from diluted mimic patient blood with slightly different color and distinguished cell suspension volume. (**B**) Schematic diagram of the triplet-microchannel spiral microfluidic chip interconnected with numerable tilted slits (Spiral-Slits Chip) for separation of tumor cells.

**Figure 4:**
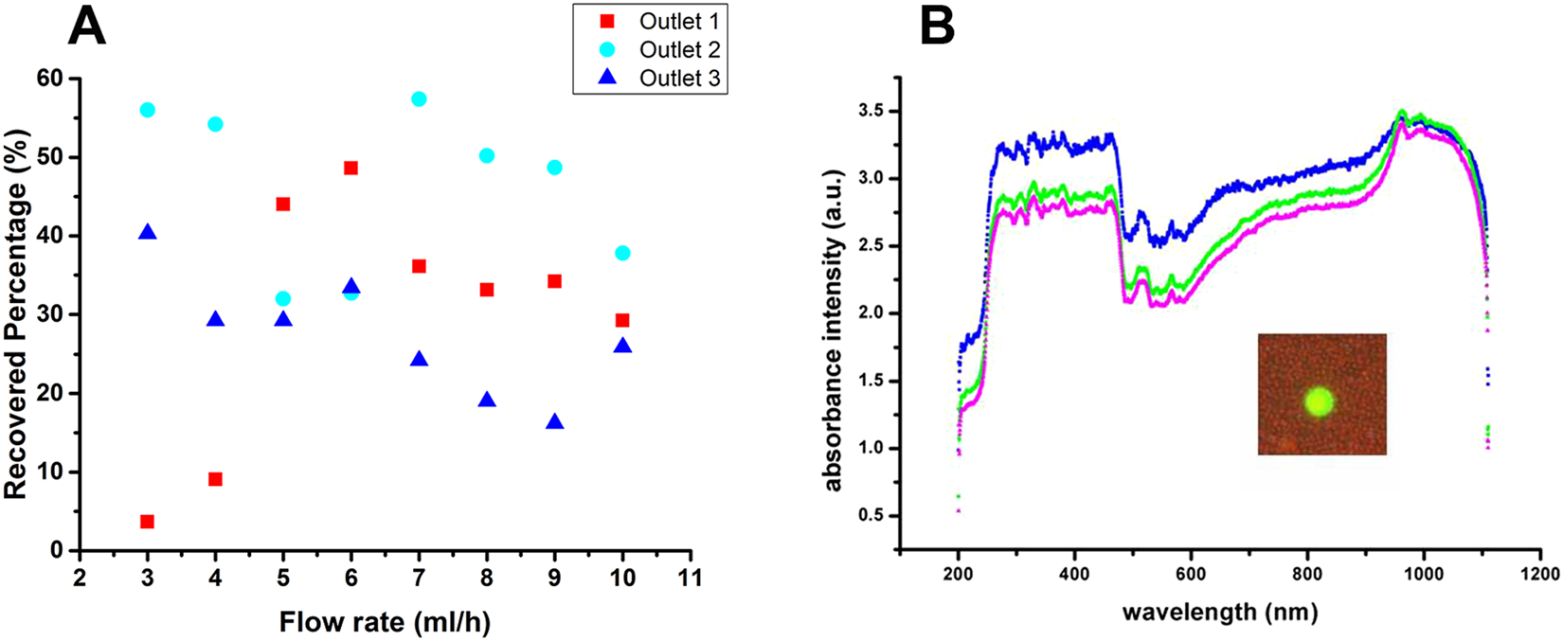
Recovered percentage and optical spectroscopy (**A**) Recovered percentage of MCF-7 cells from three microchannels at frow rates ranging from 3 ml/h to 10 ml/h. (**B**) Optical absorption spectroscopy for three samples recovered from three microchannels at 7 ml/h (blue from microchannel 2, green from microchannel 1 and red from microchannel 3). Insert: Blood sample recovered from the outlet with one MCF-7 cell stained with Calcein AM with green fluorescence among blood.

Around one thousand MCF-7 cells were spiked into the diluted blood to perform the test. The separation was performed at various flow rates from 3 ml/h to 10 ml/h. Lightest color cell suspension in diluted blood indicates least RBCs contamination. From Fig. 3, it could be seen the volume of cell suspension in diluted blood from three outlets are distinct, and the colors are slightly different. Color Difference of cell suspension collected from three microchannels is trivial. Outside microchannel has more blood particles, thus has largest blood volume and color is deepest. Inversely, the inner microchannel has fewer blood particles of least RBC contamination, thus smallest blood volume and lightest color. We found there were tumor cells coming out from three microchannels and microchannel 2 had more comparing to the other two. There is some separation phenomenon of tumor cells. However, this is not desired since all of the tumor cells were not out from the same microchannel. From Fig. 4, we could see absorption spectroscopy has been measured at 7 ml/h for cell suspension collected from three microchannels. Absorption intensity is the logarithm of the ratio of incident light intensity to transmitted light intensity. Absorption intensity for microchannel 2 is higher from microchannel 1 and 3, and obviously, this could be seen. Every material has its own characteristic spectroscopy. Absorption spectroscopy is almost same for three cell suspension collected from three outlets since they all contain the same material of tumor cells and hematological cells.

### Recovered percentage at higher flow rates

**Figure 5:**
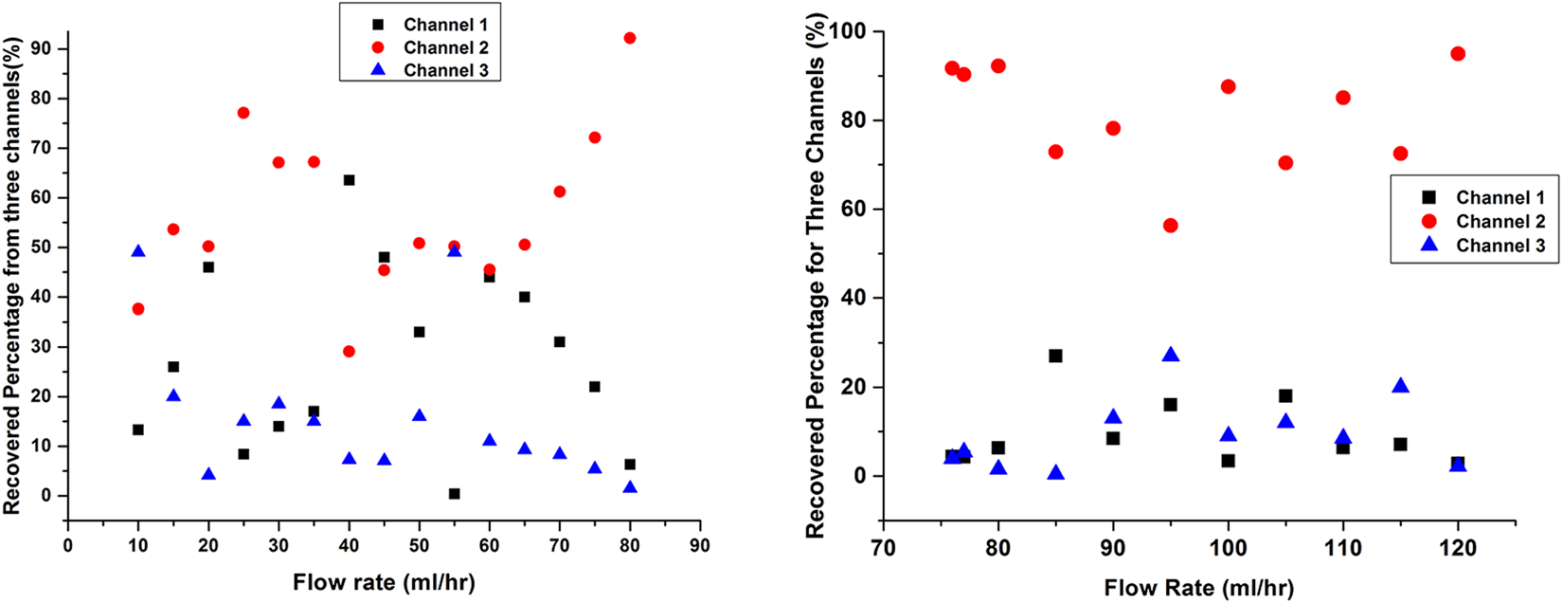
Separation of tumor cells and percentage recovered in the diluted mimic patient blood (**A**) Separation of tumor cells and percentage calculation in diluted mimic patient blood at various flow rates ranging from 10 ml/h to 80 ml/h. (**B**) Separation of tumor cells and percentage calculation in diluted mimic patient blood at distinguished flow rates ranging from 75 ml/h to 120 ml/h.

This triplet-microchannel device (Spiral-Slits Chip) is especially suitably operating for high flow rate. The separation was performed at various flow rates from 10 ml/h to 120 ml/h. Tumor cells separation is becoming evident when the flow rate is above 80 ml/h. The outside microchannel is widest, and width for the three microchannels gradually narrows down. The array of slits close to inner microchannel is narrower than the array inter-connected with outer microchannels. Both arrays of slits are organized with a certain degree of around of 60° to the fluidic direction (Fig. 2). The optimal separation should be chosen as the most percentage of tumor cells occupying the microchannel, which means, tumor cells streamline out from one microchannel. As shown from Fig. 5 for flow rate from 10 ml/h to 80 ml/h, tumor cells collected from microchannel 2 is almost occupying most. However, the optimal flow rate has not been reached most often. At 80 ml/h, more than 80% tumor cells appear from channel 2, separation starts and achieve 80–90%. At higher flow rate, apparent separation is mostly achievable as shown in Fig. 5B. At 80 ml/h and 120 ml/h, 90% tumor cells concentrated in microchannel 2. 120 ml/h means 1 ml cell suspension only takes 30 seconds to accomplish, which is extremely fast and completely satisfies the requirement. Beyond 120 ml/h, effects of separation would be much more obvious. Although there are blood cells accompanying tumor cells recovered, 90% tumor cells coming out from one single microchannel. This effect could be used to substitute for RBCL. Combined with physical-based filters, tumor cells could be captured soon. RBCL have to deal with displacing on ice and centrifugation. Both could be avoided. Comparing with RBCL, no residue appears from break of RBCs to disturb detection. Combining with Ellipse filters, characterization and release captured tumor cells or CTCs with high purity could favor performing further biological molecular analysis. Secondly, based on no worse effects exists, this pre-processing take shorter to accomplish CTC pre-processing comparing with traditional RBCs, which is just a couple of minutes. Traditional pre-processing would take about half an hour. Thirdly, processing is simplified and no transfer of test tubes avoiding loss of tumor cells during this procedure.

### Detection with optical spectroscopy

**Figure 6:**
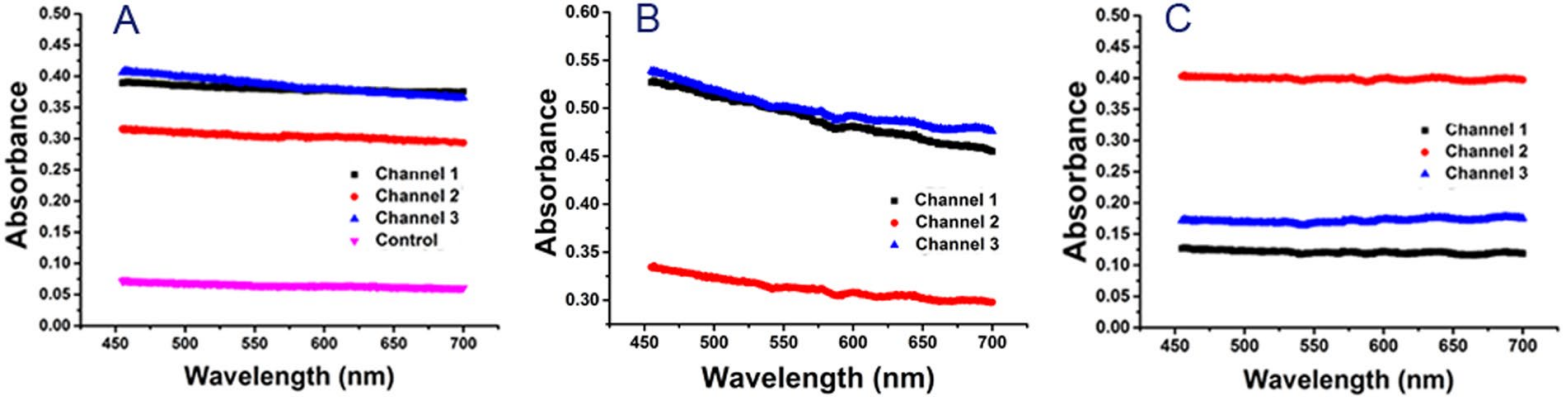
Characterization of the triplet-microchannel spiral microfluidic chip (Spiral-Slits Chip) at distinct flow rates (**A**) Optical absorption spectra for three individual cell suspension collected from three outlets at a flow rate of 25 ml/h. Control is optical absorption spectra for pure water. Absorption intensity for cell suspension coming out from microchannel 2 is obviously different absorption intensity for cell suspension coming out from microchannel 1 and 3. (**B**) Optical absorption spectra for three individual cell suspensions collected from three outlets at a flow rate of 80 ml/h. Similar phenomena happened. Absorption intensity for cell suspension coming out from microchannel 2 is distinguished from those from microchannel 1 and 3. (**C**) Optical absorption spectra for three individual cell suspension collected from three outlets at flow rate of 120 ml/h. Absorption intensity for cell suspension coming out from microchannel 2 is far away from those from microchannel 1 and 3.

About detection, we take optical absorption spectra, which is obviously different from traditional immunofluorescence. Collect cell suspension from three outlets and place them into ultraviolet spectrophotometer (Ocean Optics Co., Maya 2000/2000 PRO).Absorption intensity for cell suspension collected from microchannel 2 corresponding to middle is far from the other two at 25 ml/h, 80 ml/h and 120 ml/h in Fig. 6. Absorption spectra from microchannel 1 and 3 corresponding to inner and outer are overlapped with or close to each other. They pull down with absorption spectra from microchannel 2. Absorption intensity difference for 25 ml/h is about 0.1, 0.2 for 80 ml/h, and 0.25 for 120 ml/h, respectively. Separation could be achievable in these three situations. Separation is much better for 120 ml/h and 80 ml/h than 25 ml/h. Separation spectroscopy is different for three situations since contained material is different. Compared with traditional immunofluorescence, optical separation is intuitively with spectra immediately shown. Secondly, the staining procedure has been avoided. Without fixing and permeability, staining with stain dyn, we just collect cell suspension and place into the spectrophotometer, spectra come out directly. Thirdly, processing becomes shortly. Traditional immunofluorescence takes about 1 hour to finish staining and observation. Optical absorption spectra take only several minutes. About application, simplicity and intuition of optical absorption have more perspective and potential application.

## Conclusions

We have described a versatile triplet-microchannel spiral microfluidic chip interconnected with numerable tilted slits (Spiral-Slits Chip) for inertial separation of tumor cells highly efficiently. Combining with Dean forces and inertial forces, this centrifugal-based differential device would render big particles equilibrate close to the center of microchannel wall, and small particles gathered in the streamlines close to either side of the microchannel. Numerable tilted slits are arranged to have an interconnection on both sides of the microchannel wall. They function as “bridge” to frequently transport tumor cells into the middle microchannel and blood cells from the middle microchannel into inter-microchannel, respectively. Three staggered parallelizing microchannels are interconnected with two arrays of numerable tilted slits organized along the flow direction at a certain angle. Those numerable tilted slits could transport certain sized particles from inner streamline into microchannel inside. Therefore, separation could be achievable especially at a high flow rate. Optimal separation could be attained with minimal contamination happens at 80 ml/h when tumor cells spiked into the diluted blood. 90% tumor cells could be separated flowing out from microchannel 2. It is a passive and high efficient device for isolating and enriching tumor cells from blood samples or separation of 2–3 microparticles. Instead of traditional immunofluorescence detection, we take optical absorption spectra for three cell suspension collected. Absorption intensity is distinguished for cell suspension collected from microchannel 2 to 1 and 3. This is a tentative assay for detecting and characterizing separated tumor cells with the optical approach. Next step we would further optimize the geometry of the structure to achieve large-scale clinical samples for fast processing.
